# Application of artificial neural networks and multiple linear regression on local bond stress equation of UHPC and reinforcing steel bars

**DOI:** 10.1038/s41598-021-94480-2

**Published:** 2021-07-23

**Authors:** Ahad Amini Pishro, Shiquan Zhang, Dengshi Huang, Feng Xiong, WeiYu Li, Qihong Yang

**Affiliations:** 1 Department of Civil Engineering, Sichuan University of Science and Engineering, Zigong, China; 2 Sichuan University, Chengdu, China; 3 Southwest Jiaotong University, Chengdu, China

**Keywords:** Civil engineering, Applied mathematics, Computational science

## Abstract

We investigated the use of an Artificial Neural Network (ANN) to predict the Local Bond Stress (LBS) between Ultra-High-Performance Concrete (UHPC) and steel bars, in order to evaluate the accuracy of our LBS equation, proposed by Multiple Linear Regression (MLR). The experimental and numerical LBS results of specimens, based on RILEM standards and using pullout tests, were assessed by the ANN algorithm using the TensorFlow platform. For each specimen, steel bar diameters ($$d_{b} )$$ of 12, 14, 16, 18, and 20, concrete compressive strength ($$f_{c}^{\prime }$$), bond lengths ($$L$$), and concrete covers ($$C$$) of $$d_{b}$$, $$2d_{b}$$, $$3d_{b}$$ and $$4d_{b}$$ were used as input parameters for our ANN. To obtain an accurate LBS equation, we first modified the existing formula, then used MLR to establish a new LBS equation. Finally, we applied ANN to verify our new proposed equation. The numerical pullout test values from ABAQUS and experimental results from our laboratory were compared with the proposed LBS equation and ANN algorithm results. The results confirmed that our LBS equation is logically accurate and that there is a strong agreement between the experimental, numerical, theoretical, and the predicted LBS values. Moreover, the ANN algorithm proved the precision of our proposed LBS equation.

## Introduction

An Artificial Neural Network (ANN) can capture linear and non-linear relationships between statistical inputs and output data models, using methods inspired by biological neural networks^1^. In fact, an ANN acts as an informational system, in order to simulate the network between many simple neurons. Each neuron receives input data from different sources and applies linear and non-linear functions or calculations to provide output results. In other words, an ANN creates patterns between a set of input values against a corresponding range of outputs^2,3^. In 1943, for the first time, McCulloch and Pitts provided a simple computational model of an ANN^4^. Since then, the use of ANNs has sharply increased and, at present, such networks are widely applied in diverse scientific areas^5–12^.

At present, ANNs are used to analyze data in different fields, such as manufacturing, business, engineering, management, and other scientific aspects^13–17^. Data analysis, such as fault tolerance, convergence, scalability, performance, and accuracy, can be applied in various research areas—especially in engineering fields—to predict or evaluate the precision of results, formulas, and computational terms^18^. In general, ANNs transmit the data and information from their input layer to the output layer. The final calculated values are the results of this transmission loop, learning from wrong-doing and right-doing, which can be called feedback. To evaluate the success and accuracy of ANN models, we apply different validation metrics and functions, such as the absolute percentage error (MAPE), mean absolute error (MAE), mean squared error (MSE), and variance of absolute percentage error (VAPE)^19–23^.

ANN covers a wide range of applications in structural engineering studies^24,25^. Yeh et al.^26^ were the first to develop a neural network model to discover the relationship between High Performance Concrete (HPC) mix design proportion and compressive strength. The inputs were cement, blast furnace slag, superplasticizer, water, fly-ash, coarse aggregate, fine aggregate, and testing age. They observed an acceptable agreement in the research by comparing their results with experimental data, as well as a statistical regression model. Mehdi Nikoo et al.^27^ performed an investigation on prediction of concrete compressive strength using an evolutionary ANN. They proposed an ANN model using a non-linear specialty to determine the compressive strength of concrete. In their study, 173 experimental data patterns of cylindrical concrete specimens were used. Sakshi Gupta^28^ studied the ANN applications to predict the compressive strength of concrete containing nano-silica; an ANN model with correlation coefficient of 0.8685 was developed. An ANN can provide logical predicted values of compressive strength for 28 day-old specimens. Suryadi et al.^29^ conducted a research work on the usage of an ANN to evaluate the compressive strength of Self Compacting Concrete (SCC). In their research, the SCC mix proportions were the input values of the neural network, while the compressive strength of 28-days specimens was the output parameter. After training and testing their neural network, they found a reasonable prediction of the ANN, compared to their experimental results, with an error of 10%. Muthupriya et al.^30^ employed feed-forward NNs to determine the compressive strength of HPC specimens at the ages of 3, 7, 28, 56, and 90 days. They also investigated the durability of HPC containing silica fume and metakaolin as additives. Their comparison showed that the experimental results could be coherently predicted by ANNs. They also found ANNs more applicable methods for analyzing unstructured non-linear problems, compared to the general models of mathematical regression. Researchers have used neural networks to study different properties of concrete, such as saturated water absorption, acid resistance, porosity, and permeability. One of the most constructive additives to enhance the compressive strength of concrete is nano-silica. This material is a super effective pozzolanic material consisting of extremely fine particles. The small size of the nano-silica particles and their chemical adhesion made them practical admixture to reduce the weakness points of concrete (e.g., permeability) and improve properties such as compressive strength and durability^31^. Gupta et al.^28^ performed a study on the effect of nano-silica addition to increase the concrete compressive strength ($f_{c}^{\prime }$). They collected their experimental results from specimens at the age of 28-days and, then, applied an ANN model to predict the $f_{c}^{\prime }$ values. After training and testing, they obtained a good agreement between their experimental and numerical results. They indicated that the effect of each principal material or additive on concrete behavior can be logically evaluated by the ANN methodology. The research works of Abellán et al.^32–34^ have focused on combining different methods of neural networks, data mining, multi-objective optimization, and design of experiments (DOE). They studied how to improve the compressive strength over 150 MPa by using local raw materials in Colombia, while reducing the content of silica admixture. The aim was to decrease the final cost of making UHPC. They found that acceptable mechanical properties could be obtained by replacing silica-based additives with limestone and recycled glass powder.

Multiple linear regression (MLR) analysis according to least-squares procedures is normally applied to estimate model equation coefficients. Many researchers have conducted studies on UHPC materials, the effects of additives on concrete durability, and compressive strength. Charhate et al.^35^ used ANN and MLR techniques to predict concrete slump, and compressive strength of 28 day-old concrete. Water, fine aggregate, coarse aggregate, and cement, were the parameters in that study. The investigation showed that their proposed model by MLR, delivers good results. Additionally, Khademi and Behfarnia^36^ evaluated concrete compressive strength using ANN and MLR models with a correlation coefficient above 0.9 and under 0.9, respectively. The results demonstrated that their model is superior in comparison to expensive experimental tests.

Among all of the previous research works^37–50^, the applications of ANN and MLR methods to predict and determine the mechanical interactions between concrete and steel bars, such as local bond stress (LBS), have not been covered. Therefore, in this study, we developed the use of an ANN algorithm in structural engineering by checking the accuracy of our LBS equation, proposed by MLR. We conducted a comprehensive investigation on the local bond stress between UHPC and steel bars^51^. In this research, 144 specimens were made, based on RILEM standards^52^. Sixteen of these experimental specimens, as well as 39 new ones, were modeled using the finite element software ABAQUS, in order to conduct our numerical investigation. MLR technique was carried out to derive the most accurate LBS equation model. The values from experimental and numerical results were compared to the values of our proposed LBS equation. The parameters of concrete cover $\left( C \right)$, steel bar size $\left( {d_{b} } \right)$, bond length $\left( L \right),$ and concrete compressive strength $\left( {f_{c}^{\prime } } \right)$ of our UHPC specimens served as input values to the ANN. Since LBS is the critical local bond stress between steel bar and concrete, we set the range of validity for bond length from $1{\text{D}}$ to $4{\text{D}}$. The output layer was comprised of our experimental and numerical local bond stress $\left( {u_{c} } \right)$ results, as well as the values obtained from our proposed LBS equation. After training and testing the ANN and conducting a fair comparison between all of the experimental, numerical, and theoretical results, the accuracy of our LBS equation was proved. This equation, therefore, can be used by all engineers and researchers to calculate the local bond stress between UHPC and steel bars.

## Data acquisition

The input values and features of our ANN algorithm were the effective parameters of the local bond stress between UHPC and steel bars, including Steel bar size ($R$), concrete cover ($C$), bond length ($L$), and Concrete compressive strength ($f_{c}^{\prime }$). Local bond stress values, resulting from our experimental pullout tests and numerical studies conducted by ABAQUS, were assigned as the output of the ANN. After training and testing the ANN, we compared the predicted outcomes with the experimental, numerical, and LBS equation results. The aim was for the ANN to investigate the relationship between $R, C, L, \;{\text{and}}\;f_{c}^{\prime }$ and the local bond stress, $u_{c}$, in order to evaluate the accuracy of our proposed LBS equation.

**Figure 1:**
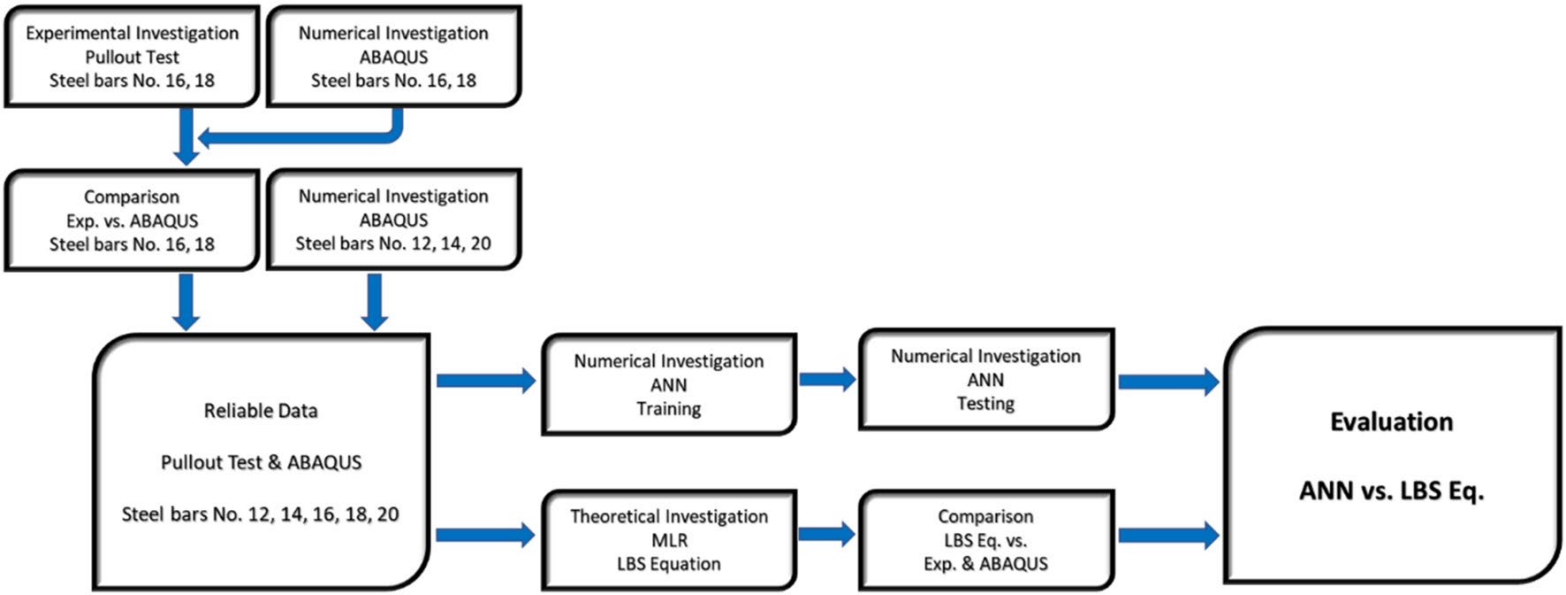
Research structure.

As Fig. 1, research structure presents, there were different evaluation and comparison steps in our research plan. Experimental data were compared to the numerical results. After checking the consistency of our experiments in the lab, we conducted a theoretical study to modify the existing LBS equation. Reliable data from specimens with steel bar Nos. 12, 14, 16, 18, and 20 were used to assess our proposed LBS equation. Training the ANN and conducting further comparative steps were done using our reliable data source. This comprehensive investigation ended in a fair assessment, proving the consistency of the results of this research work.

### Experimental program

To investigate the Local Bond Stress (LBS) between UHPC and steel bars, 144 specimens were made, based on RILEM standards^52^. Steel bar size, concrete cover, bond length, and concrete compressive strength were the parameters considered when designing these specimens. To increase the UHPC compressive strength, nano-silica was used as an additive to the mix design, by 6.5% of cement weight. Table 1 presents the UHPC mix design used in this research. In this paper, we used the fourth mix design (containing 6.5% of nano silica content) to reach the desired UHPC compressive strength of 155 MPa for our research.

**Table 1:** UHPC mix designs (kg/m^3^)^53^.

Design no	Cement	Quartz sand	Quartz powder	Micro silica	Nano silica	Water	Super plasticizer
1	665	1020	285	200	0	178	23
2	665	1020	285	183.375	16.625	178	23
3	665	1020	285	170.075	29.925	178	23
4	665	1020	285	156.775	43.225	178	23

After demolding the specimens, some of them were kept under thermal curing at 60 °C and 95% moisture for 72 h, whereas the other specimens were kept in a 20 °C water tank for 28 days. It has been found that thermal curing increased the UHPC compressive strength by more than 40%^51^.

In this study, the specimens were named in the format R20C2L3F155. R20 means that the reinforcing steel bar No. 20 was used to make this specimen. The letter C stands for the concrete cover on the steel bar, L shows the bond length, and the letter F indicates the UHPC compressive strength (in MPa)^51^. As mentioned above, the concrete covers were considered in the four different sizes of $d_{b}$, $2d_{b}$, $3d_{b}$, and $4d_{b}$ where $d_{b}$ represents the steel bar diameter (in mm). So, in the R20C2L3F155 specimen, C2 indicated the second concrete cover $\left( {2 \times 20 = 40\;{\text{ mm}}} \right)$, whereas L3 belonged to the third type of bond length $\left( {3 \times 20 = 60 \;{\text{mm}}} \right)$. F155 represents the concrete compressive strength, $f_{c}^{\prime } = 155\;{\text{MPa}}$.

**Figure 2:**
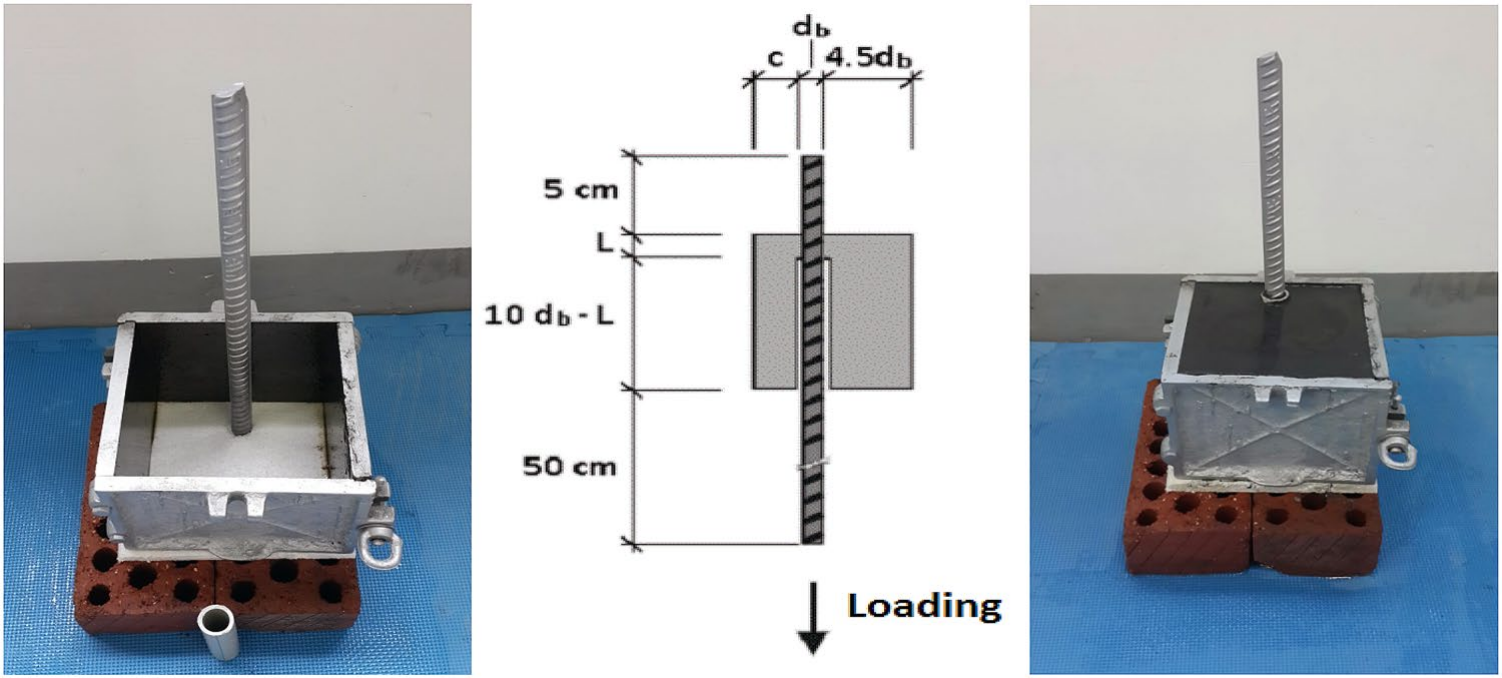
Geometric dimensions of RILEM standard specimens^51^.

Figure 2 shows the geometric characteristics of the specimens, based on RILEM standards^51^.

**Figure 3:**
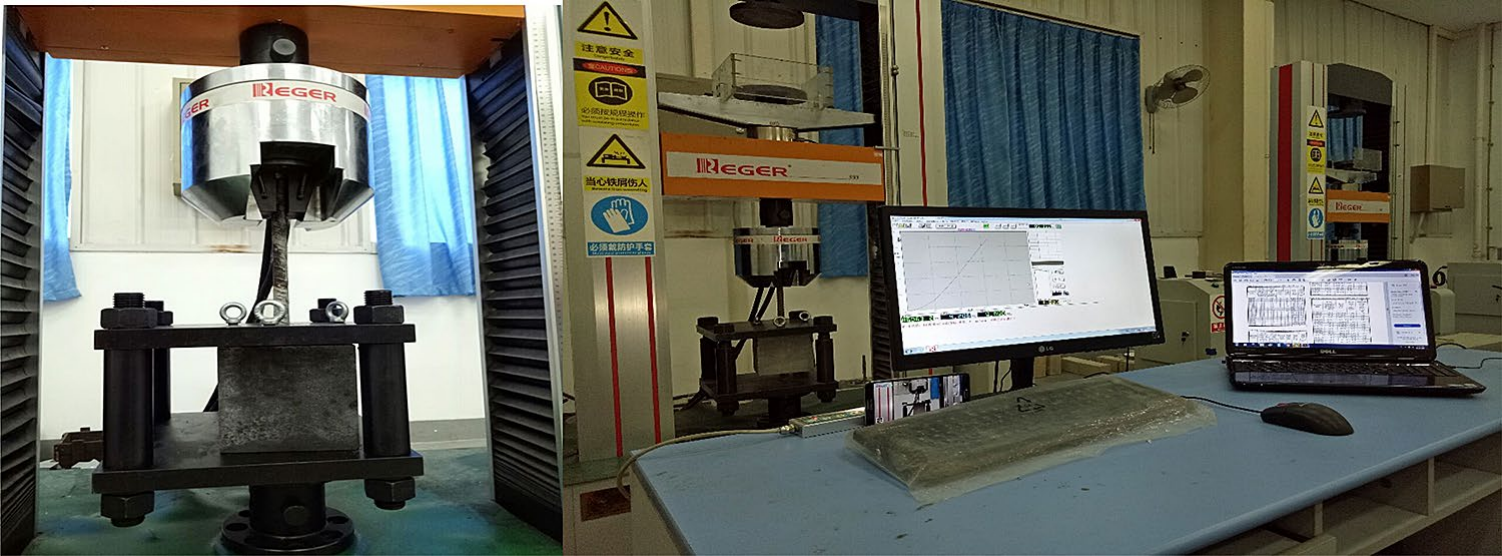
Pullout test lab and specimen installation^51^.

American Standard for Testing and Material, ASTM C234-91a, proposed the pullout test as the most practical experiment to investigate the bond stress between concrete and steel bars^52,54,55^. Figure 3 presents the pullout test laboratory and the location of specimens and grip under the jack.

### Numerical program

**Figure 4:**
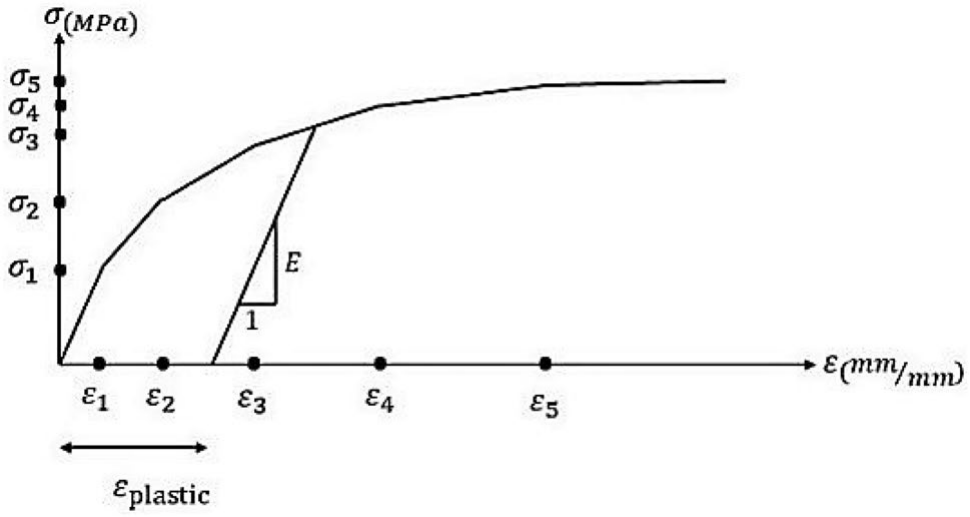
Non-linear model for stress–strain curve of concrete; compressive behavior.

**Figure 5:**
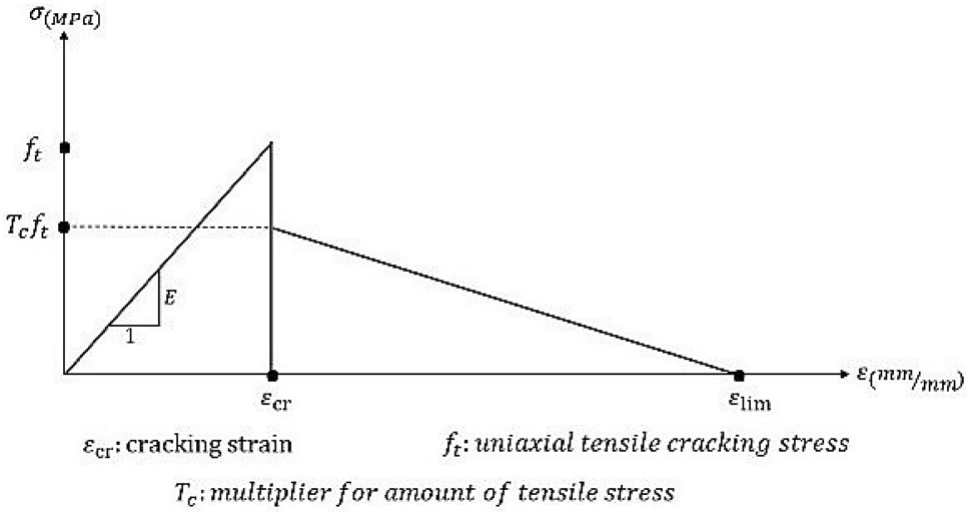
Non-linear model for the stress–strain curve of concrete under tension.

ABAQUS, a finite element software^56^, was used to verify the experimental results of specimens R16 and R18. To this aim, after modeling our specimens in ABAQUS and running the concrete damage plasticity model, the obtained LBS values were compared with the results of our experiments. To complete the numerical studies, and considering the expense of experimental studies, specimens of steel bar sizes 12, 14, and 20, including different concrete covers and bond lengths from $d_{b}$ to $4d_{b}$, and of concrete compressive strength 155.1 MPa, were analyzed by ABAQUS. To conduct a precise numerical analysis of RC structures in ABAQUS, we had to determine an appropriate non-linear behavior for concrete. To do so, $\sigma_{1}$ was assigned (as the stress) to $0.45f_{c}$, $E$ (for modulus of elasticity) equal to $4700\sqrt {f_{c} }$, and $\varepsilon_{1}$ (for strain)^57^. Figure 4 presents the stress–strain curve of non-linear compressive behavior in concrete. Moreover, the non-linear model of concrete behavior under tension is illustrated in Fig. 5, where $\varepsilon_{cr}$ indicates the strain corresponding to the maximum tensile stress of concrete $\left( {f_{t} = 0.55\sqrt {f_{c} } } \right)$.

**Figure 6:**
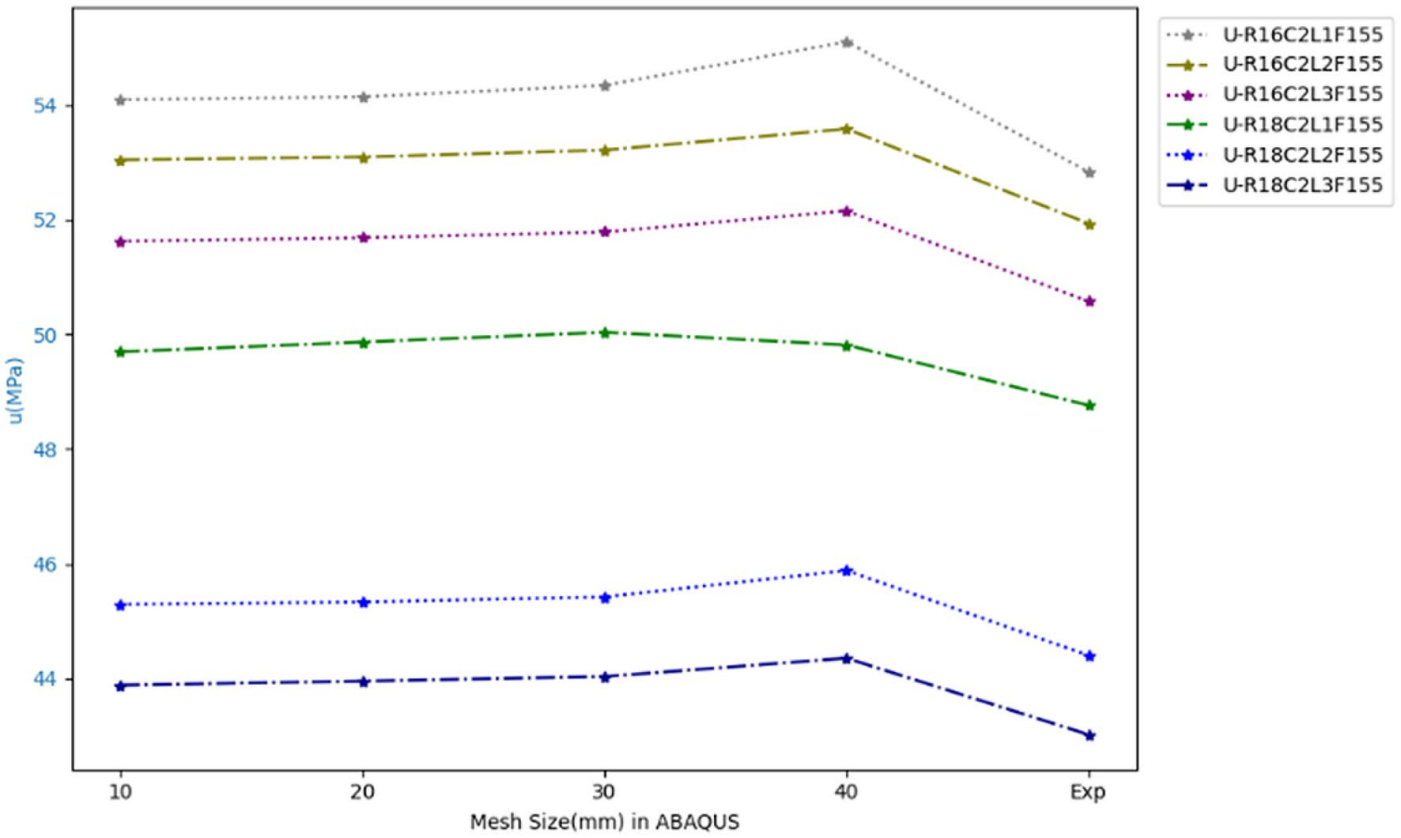
Bond force and bond stress results from ABAQUS with different dimensions of elements, compared to the experimental results.

To select the most consistent mesh size for our specimens in ABAQUS, we compared the experimental values with the numerical results obtained from different element dimensions of 10, 20, 30, and 40 mm. Figure 6, Bond force and bond stress results from ABAQUS, shows that the difference between our experimental and numerical results, when using the mesh size of 20 mm in ABAQUS, was about 2%. Therefore, to meet the accuracy requirements of our study, we chose the size of 20 mm for our elements^51^.

**Figure 7:**
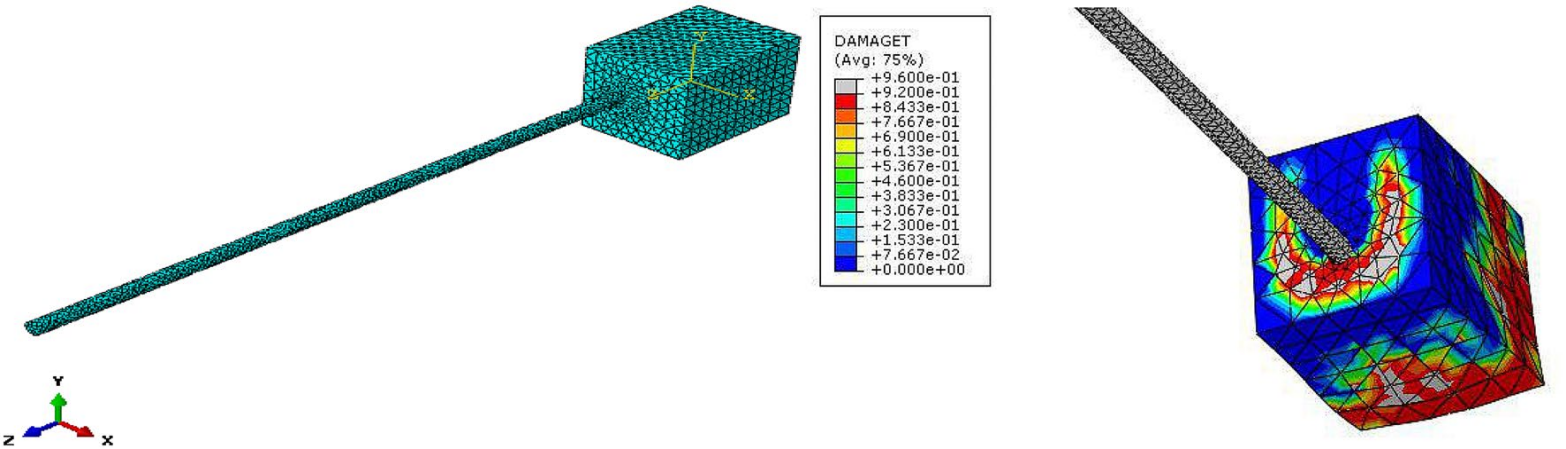
Specimen modeling in ABAQUS.

The bond stress between a steel bar and UHPC is not distributed constantly along the bond length. In this study, we used the simple equation $\tau_{av} = \frac{P}{\pi dl}$ to calculate the average bond stress, which is indicated by $\tau_{av}$. The bond length is shown by $l$, while $d$ represents the steel bar diameter. The bond force is represented by $P$. Figure 7 shows the modeling of specimens in ABAQUS. As can be seen, the finite element method was conducted to analyze the effects of steel bar size, bond length, concrete cover, and compressive strength on local bond stress. This analysis was started from initial loading and ended with specimen failure.

## Equation proposal and application of ANN

### Pullout test and ABAQUS

Based on the research plan, we conducted a comparison between our experimental and numerical results, in order to investigate the effects of $R, C, L, \;{\text{and}}\;f_{c}^{\prime }$ on the local bond stress $u_{c}$^51^. With regards to the equation $\tau_{av} = \frac{P}{\pi dl}$, increasing the bond length decreases the local bond stress; however, concrete cover has an effect to the contrary. We found that LBS increased with increasing $C$. In fact, the crack spacing was greater with higher concrete covers, compared to small covers. Therefore, the number of cracks around the steel bars decreased from $C1$ to $C2$. This matter led to higher initial cracking force, as well as LBS values. Moreover, there was an inverted relationship between steel bar size and LBS. A greater bond surface between the steel bar and UHPC, due to an increase in the steel bar diameter, resulted in decreased LBS. As mentioned above, we added nano-silica by 6.5% of cement weight. This additive improved the concrete compressive strength to 155 MPa. Nano-silica enhanced the microstructure of UHPC and, by filling the space between the steel bar surface and the surrounding concrete, we obtained higher $u_{c}$ values.

**Figure 8:**
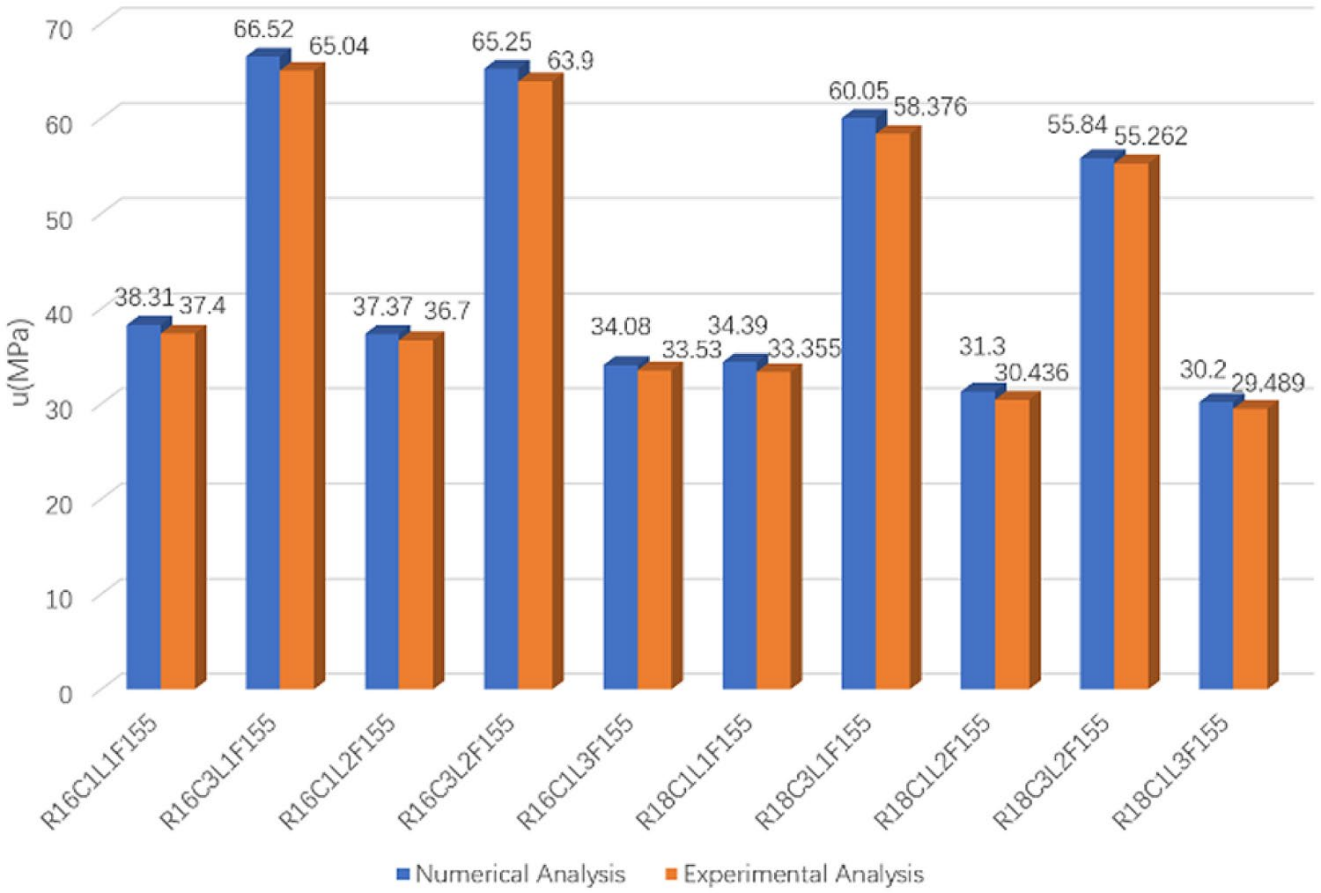
Bond stress results: numerical analysis vs. experimental analysis.

Figure 8, Bond stress results, presents a comparison of some random specimens between experimental (pullout test) and numerical (ABAQUS) studies. It can be seen that experimental pullout test results were logically consistent with the results from ABAQUS.

Knowing that experimental results are time- and cost-consuming, we extended our numerical investigation to a comprehensive parametric study. In this part, concrete cover $C4$ and bond length $L4$, including other, more commonly used steel bars (Nos. 12, 14, and 20) were used to model our specimens in the finite element software ABAQUS. As above, we observed the same trend of increasing or decreasing the LBS by changing the effective parameters on local bond stress. Table 2 provides the numerical LBS values of some specimen results from ABAQUS.

**Table 2:** Numerical LBS results from ABAQUS.

Specimen	*C* (mm)	*L* (mm)	*f*_*c*_ (Mpa)	*f*_*ct*_ (Mpa)	Bond force (kg)	*u*_Num._ (Mpa)
R12C4L1F155	48	12	155.1	6.85	3949.75	85.65
R12C4L2F155	48	24	155.1	6.85	7644.68	82.89
R12C1L4F155	12	48	155.1	6.85	6972.96	37.80
R14C4L1F155	56	14	155.1	6.85	4977.83	79.31
R14C4L2F155	56	28	155.1	6.85	9634.50	76.75
R14C1L4F155	14	56	155.1	6.85	8787.94	35.00
R16C4L1F155	64	16	155.1	6.85	6239.49	76.11
R16C4L2F155	64	32	155.1	6.85	12,059.51	73.55
R16C1L4F155	16	64	155.1	6.85	10,979.08	33.48
R18C4L1F155	72	18	155.1	6.85	6878.00	66.29
R18C4L2F155	72	36	155.1	6.85	13,272.02	63.96
R18C1L4F155	18	72	155.1	6.85	11,747.93	28.31
R20C4L1F155	80	20	155.1	6.85	7782.97	60.76
R20C4L2F155	80	40	155.1	6.85	15,074.39	58.84
R20C1L4F155	20	80	155.1	6.85	13,411.94	26.18

According to the test results, three main failure modes were recognized: Split, Pullout, and Bar yielding. In split mode, hoop tensile stress reached the tensile strength of concrete; failure of the specimen was accompanied with wide radial cracking and splitting into two or more sections. In pullout mode, the reinforcing bar was pulled out of the concrete by reducing the interaction between concrete and the steel bar to the maximum shear capacity of concrete. In this case, concrete remained intact without any cracks or complications indicating destruction. This failure mode was observed in specimens with thick cover. Increase in concrete strength was the other factor which resulted in the pullout mode. This is due to an increase in the tensile strength of the concrete surrounding the reinforcing bar, which leads to predominant shear failure (pullout mode). In other words, by nano-silica addition, the failure mode changes from split to pullout. Finally, the bar yielding failure mode occurs because of the long development length or high strength of concrete. In this case, the reinforcing bar yields and splits before the bond zone reaches the ultimate capacity. These three different failure modes were observed in experimental and numerical tests^51^.

### LBS equation

According to the ACI 408 Committee^58^, there exist five equations to calculate the bond stress between concrete and a steel bar^59,60^. Comparison of our experimental and numerical results with the existing equations proved that we needed a more precise formula to calculate the LBS. To modify the LBS equation, we conducted two different theoretical methods, in order to obtain two equations. Then, by applying an investigative comparison between our theoretical, numerical, and experimental results, we proposed the most accurate LBS equation.

For the first theoretical method, we applied our data on Esfahani and Rangan’s equation^61^, which is a modification of Tepfer’s theory:

1 $$u_{c} = 8.6\frac{{\left( {\frac{c}{{d_{b} }} + 0.5} \right)}}{{\left( {\frac{c}{{d_{b} }} + 5.5} \right)}}f_{ct} \;\;: f_{c}^{\prime } \ge 50\;{\text{MPa}}$$

In this equation, $d_{b}$ represents the steel bar diameter, $u_{c}$ indicates the bond stress, and $c$ is the minimum concrete cover. By having concrete compressive strength ($f_{c}^{\prime }$), we calculate $f_{ct} = 0.55\sqrt {f_{c}^{\prime } }$.

To simplify our modification, we define the initial value $f_{b} = \frac{{\frac{c}{{d_{b} }} + 0.5}}{1.75}f_{ct}$. Therefore, Eq. 1 can be defined as

2 $$u_{c} = 8.6\frac{{1.75f_{b} }}{{\frac{c}{{d_{b} }} + 5.5}}f_{ct}$$

Equation 2 can be modified by assigning two constant coefficients, $c_{1}$ and $c_{2}$, as follows:

3 $$u_{c} = 1.75\left( {c_{1} \times \frac{{f_{b} }}{{\frac{c}{{d_{b} }} + c_{2} }}} \right),$$

4 $$\frac{c}{{d_{b} }} = 1.75 \times c_{1} \times \left( {\frac{{f_{b} }}{{u_{c} }}} \right) - c_{2}$$

**Figure 9:**
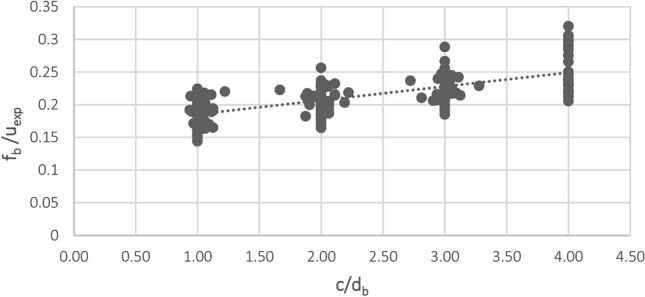
Linear relationship: $\frac{{f_{b} }}{{u_{test} }}$ vs. $\frac{c}{{d_{b} }}$.

Figure 9, Linear relationship between $\frac{{f_{b} }}{{u_{test} }}$ and $\frac{c}{{d_{b} }}$, presents the linear relationship between the values $\frac{{f_{b} }}{{u_{test} }}$ and $\frac{c}{{d_{b} }}$ for steel bars of Nos. 12, 14, 16, 18, and 20, according to the experimental and numerical results. We obtained the following linear fitted curve, based on our data:

5 $$\frac{{f_{b} }}{{u_{c} }} = 0.0213\frac{c}{{d_{b} }} + 0.1643$$

This method led our first LBS equation to calculate the local bond stress between reinforcing steel bars and UHPC to be:

6 $$u_{c} = 26.8276\frac{{\frac{c}{{d_{b} }} + 0.5}}{{\frac{c}{{d_{b} }} + 7.7136}}f_{ct} \;\;: f_{c}^{\prime } \ge 80\;{\text{MPa}}$$

### A new equation proposed by multiple linear regression

**Figure 10:**
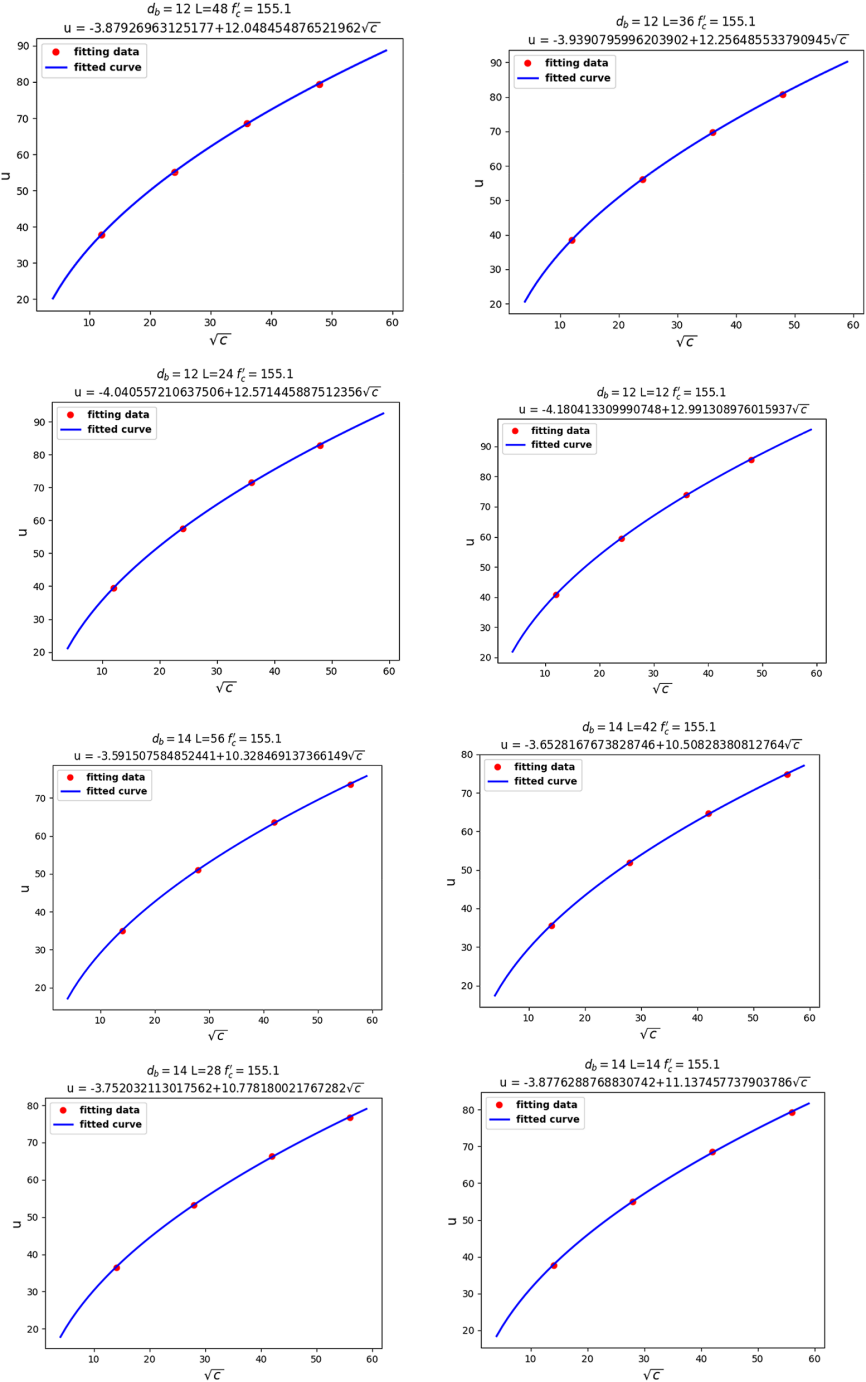

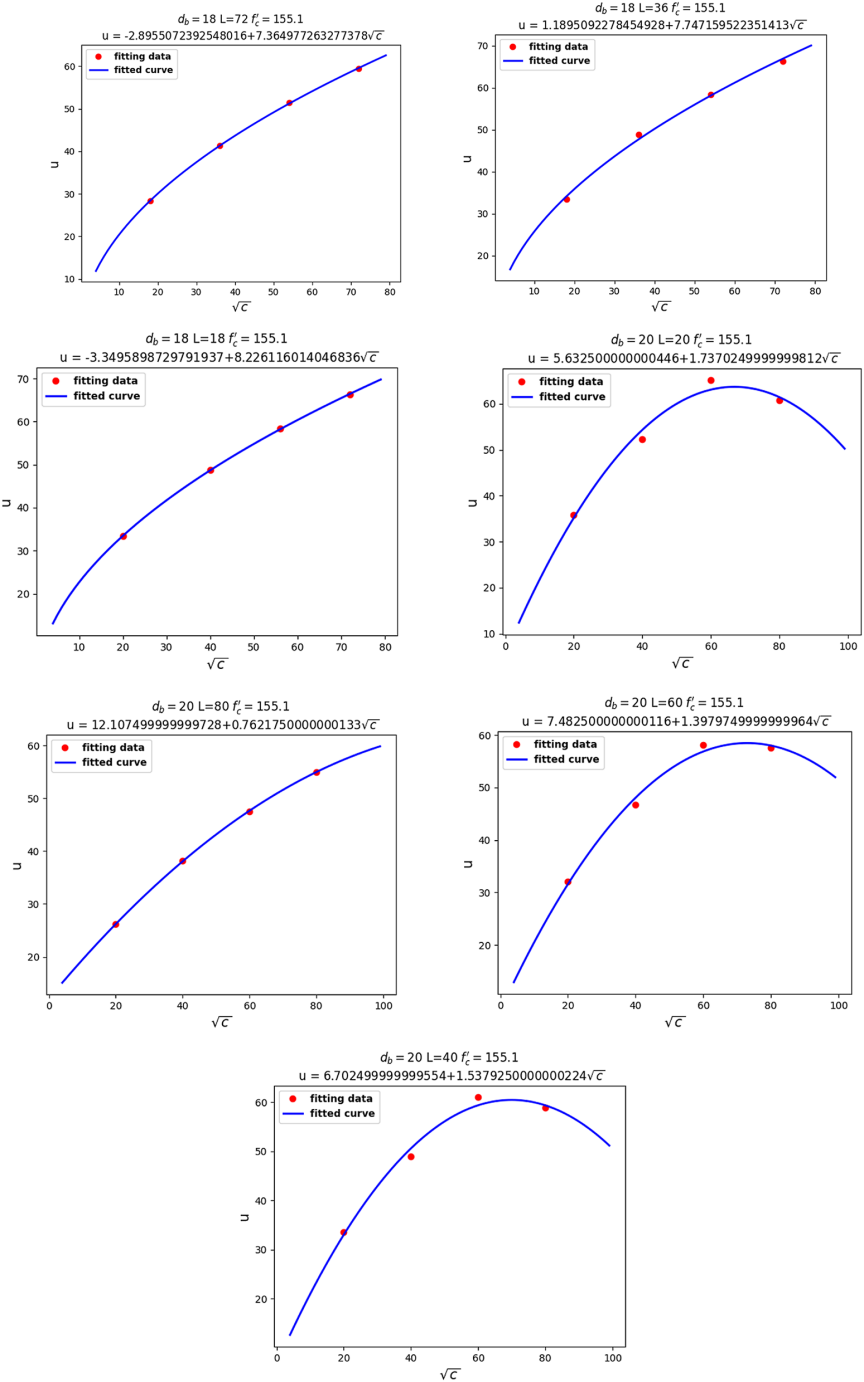
Linear relationship between $u$ and $c$.

Our second theoretical method aimed to determine the relationship between the local bond stress, $u$, and the four variables $c, d_{b} , l,$ and $f_{c}^{\prime }$. We assumed three of our four variables to be fixed in different steps, in order to observe their functional relationships. First, we considered $d_{b} , L,$ and $f_{c}^{^{\prime}}$ to be fixed and investigated the relationship between $u$ and $c$. As we can see from Fig. 10, there was a linear relationship between $u$ and $\sqrt c$.

**Figure 11:**
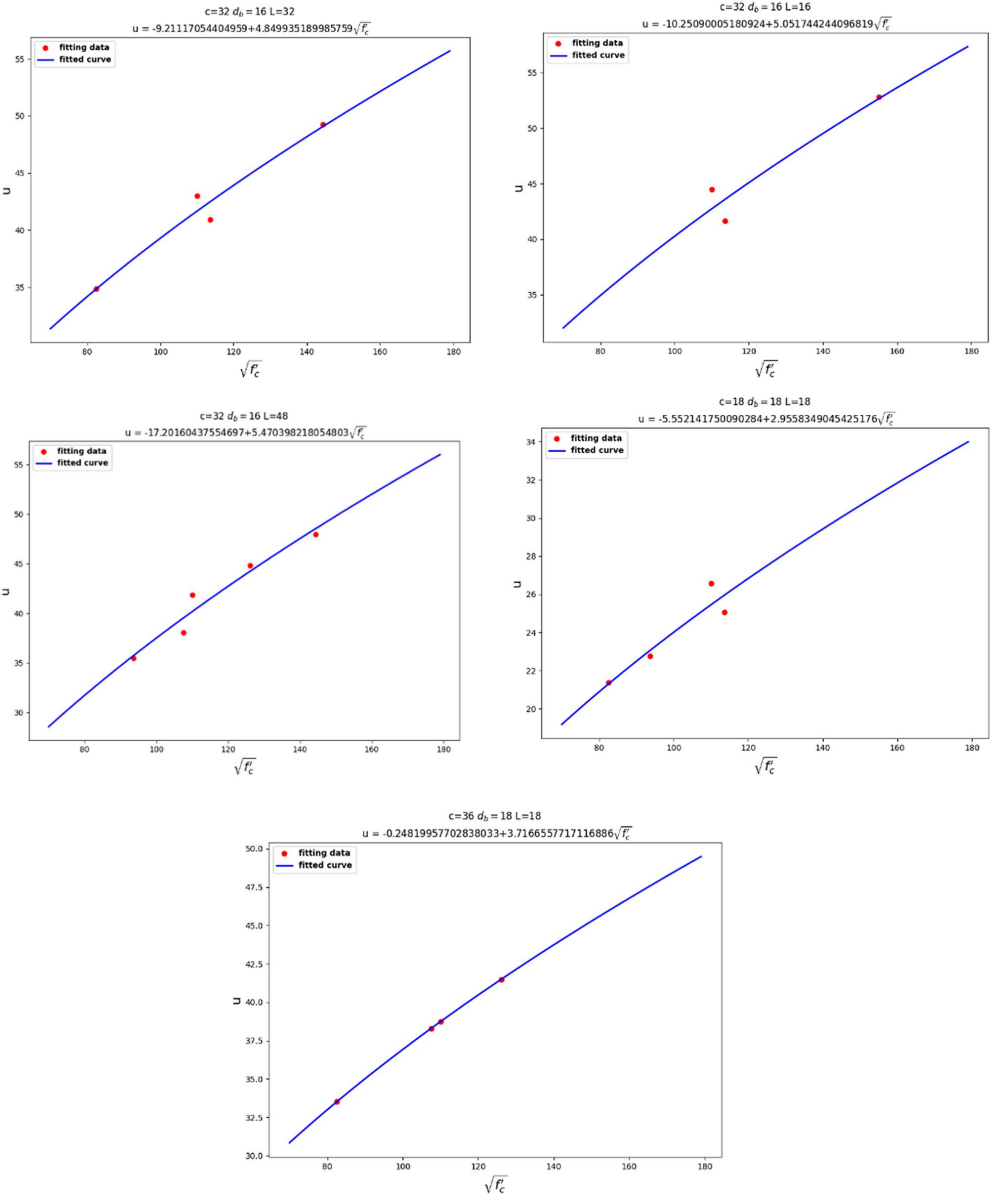
Linear relationship between $u$ and $\sqrt {f_{c}^{\prime } }$.

For the next step, as shown in Fig. 11, Linear relationship between $u$ and $\sqrt {f_{c}^{\prime } }$, we assumed our fixed parameters to be $c$, $d_{b}$, and $l$, in order to observe the relationship between $u$ and $f_{c}^{\prime }$. It can be seen that $u$ had a linear relationship with $\sqrt {f_{c}^{\prime } }$. Knowing that $f_{ct} = 0.55\sqrt {f_{c}^{^{\prime}} }$ represents the tensile stress of concrete, the linear relationship between $u$ and $\sqrt {f_{c}^{\prime } }$ seems reasonable.

**Figure 12:**
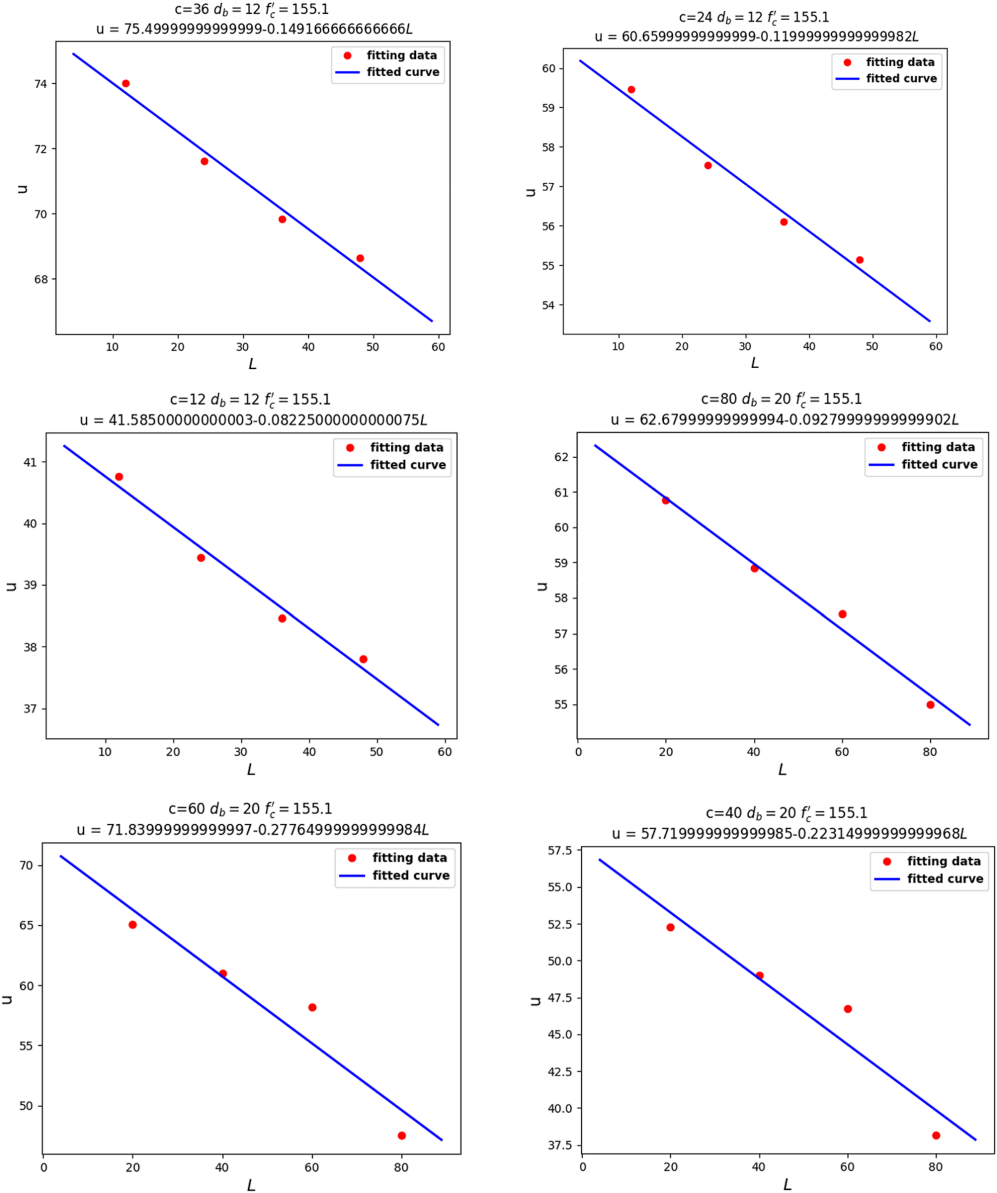

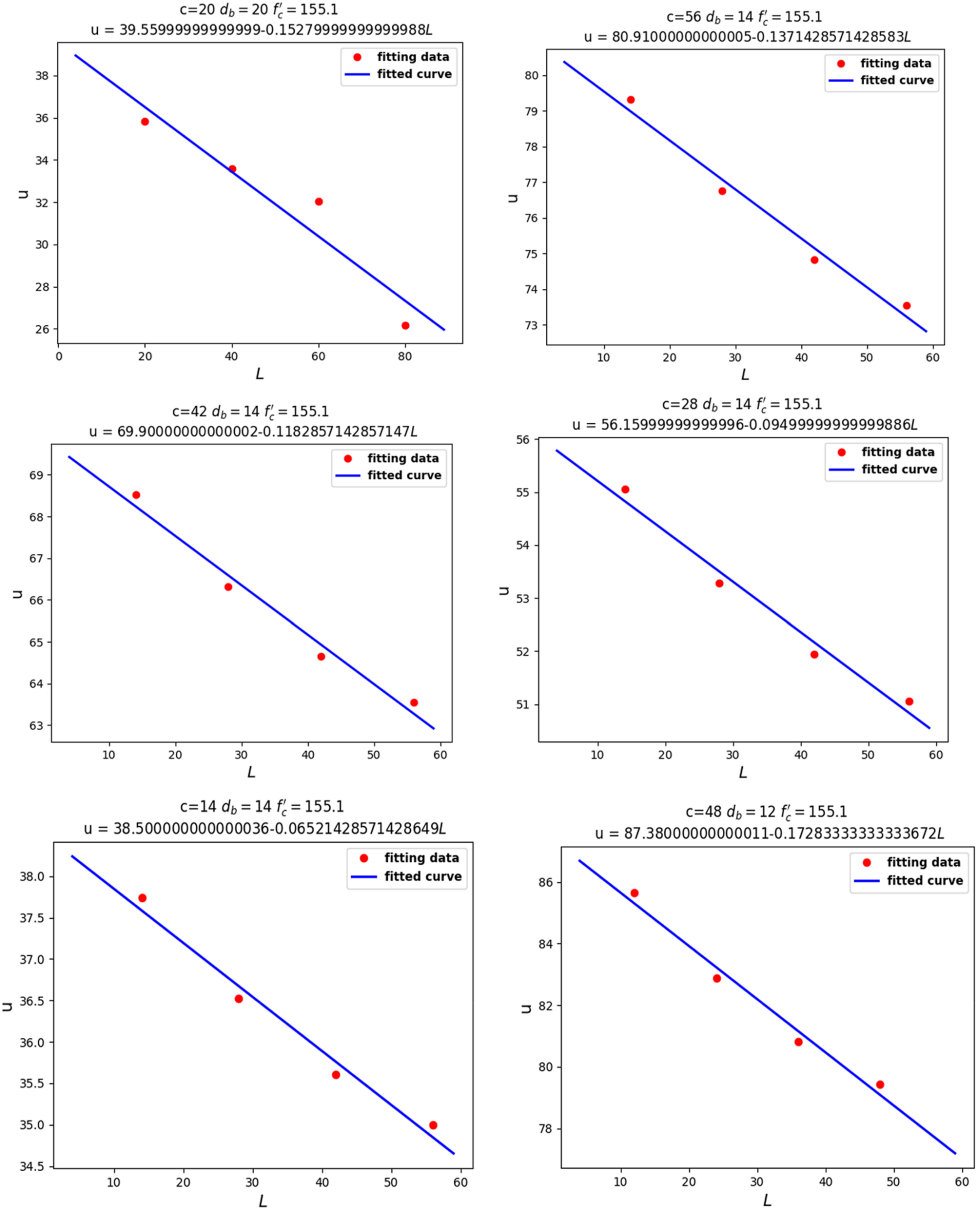
Linear relationship between $u$ and ${ }l$.

Finally, we fixed $c$, $d_{b}$, and $f_{c}^{\prime }$ to investigate the relationship between $u$ and $l$. From Fig. 12, Linear relationship between $u$ and ${ }l$, we can analyze that $u$ had a linear relationship with $l$.

Knowing the relationships between $u$, $c$, $f_{c}^{\prime }$, and $l$, we assume the equation:

7 $$u = a_{1} \frac{{\sqrt {{\text{c}}f_{c}^{\prime } } }}{{d_{b} }} + a_{2} \frac{L}{{d_{b} }}\sqrt {f_{c}^{\prime } }$$

If we assign $x_{1}$ and $x_{2}$ as follows:

8 $$x_{1} = \frac{{\sqrt {{\text{c}}f_{c}^{\prime } } }}{{d_{b} }},$$

9 $$x_{2} = \frac{L}{{d_{b} }}\sqrt {f_{c}^{\prime } } ,$$

then the following linear equation can be defined:

10 $$u = a_{1} x_{1} + a_{2} x_{2}$$

Based on known data, we can solve $a_{1}$ and $a_{2}$ by linear regression of multiple variables using machine learning techniques.

Multivariable linear regression is mainly used to study the relationship between a factor variable and multiple variables, similar to the principle of univariate linear regression. The difference is that there are more influence factors (arguments).

In statistics, linear regression equations are the product of a kind of regression analysis that uses the least square function to model the relationship(s) between one or more arguments. This function is a linear combination of one or more model parameters, called regression coefficients.

For n-dimensional feature sample data, if we decide to use linear regression, the corresponding model would be:

11 $$h_{\theta } \left( {x_{1} ,x_{2} , \ldots ,x_{n} } \right) = \theta_{0} + \theta_{1} x_{1} + \cdots + \theta_{n} x_{n}$$

This represents a simplified one and we add a feature, $x_{0} = 1$, such that

12 $$h_{\theta } \left( {x_{0} ,x_{1} ,x_{2} , \ldots ,x_{n} } \right) = \mathop \sum \limits_{i = 0}^{n} \theta_{i} x_{i}$$

Further representation in matrix form is more concise, as follows:

13 $$h_{\theta } \left( X \right) = X\theta ,$$

where

14 $$\theta = \left[ {\begin{array}{*{20}c} {\theta_{0} } \\ {\theta_{1} } \\ {\begin{array}{*{20}c} \vdots \\ {\theta_{n} } \\ \end{array} } \\ \end{array} } \right] ,\;{\varvec{x}} = \left[ {\begin{array}{*{20}c} {x_{0} } \\ {x_{1} } \\ {\begin{array}{*{20}c} \vdots \\ {x_{n} } \\ \end{array} } \\ \end{array} } \right],\;Y = \left[ {\begin{array}{*{20}c} {y_{0} } \\ {y_{1} } \\ {\begin{array}{*{20}c} \vdots \\ {y_{n} } \\ \end{array} } \\ \end{array} } \right],\;X = \left[ {\begin{array}{*{20}c} {x_{01} } & \cdots & {x_{0n} } \\ \vdots & \ddots & \vdots \\ {x_{m1} } & \cdots & {x_{mn} } \\ \end{array} } \right]$$

$m$ represents the number of samples, and $n$ represents the number of sample features.

To obtain the model, we need to find the desired loss function; generally, for linear regression, we use the mean square error as the loss function. The algebraic equation for the loss function is expressed as follows:

15 $$J\left( {\theta_{0} ,\theta_{1} ,\theta_{2} , \ldots ,\theta_{n} } \right) = \mathop \sum \limits_{i = 0}^{m} \left( {h_{\theta } \left( {x_{0} ,x_{1} ,x_{2} , \ldots ,x_{n} } \right) - y_{i} } \right)^{2}$$

The loss function, in matrix form, is as follows:

16 $$J\left( \theta \right) = \left( {X\theta - Y} \right)^{T} \left( {X\theta - Y} \right)$$

By parameter estimation of the linear regression model, in order to minimize the loss function, it is necessary to guide the following formula:

17 $$\frac{\partial J\left( \theta \right)}{{\partial \theta }} = 2X^{T} X\theta - 2X^{T} Y$$

Then,

18 $$\theta = \left( {X^{T} X} \right)^{ - 1} X^{T} Y$$

Thus, we get the following multilinear regression model:

19 $$f\left( x \right) = x^{T} \left( {X^{T} X} \right)^{ - 1} X^{T} Y$$

Finally, we obtain our new LBS equation:

20 $$u = 12.056\frac{{\sqrt {cf_{c}^{\prime } } }}{{d_{b} }} - 0.152\frac{L}{{d_{b} }}\sqrt {f_{c}^{\prime } }$$

### Application of ANN

**Figure 13:**
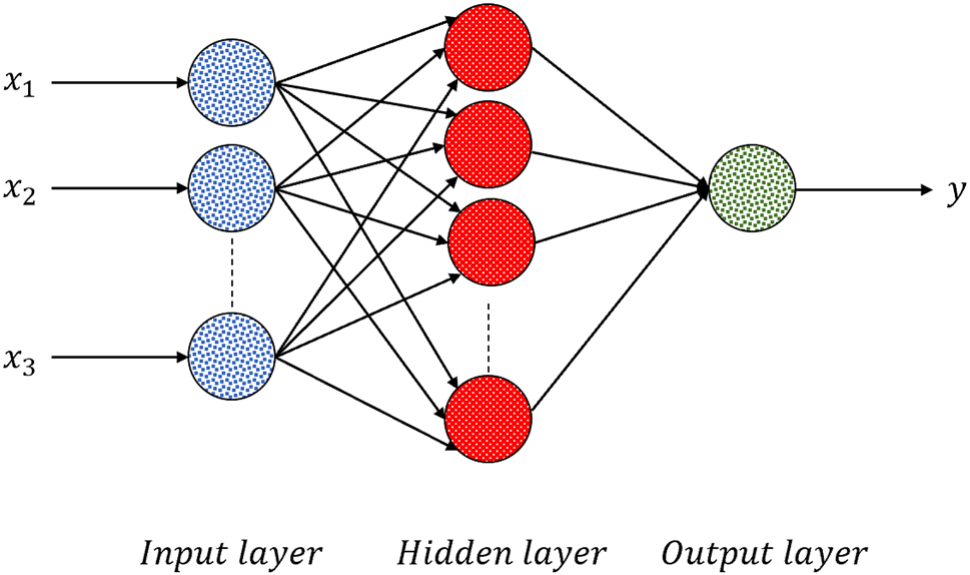
Structure of an ANN neural network.

ANN has proved to be a common functional approximation, which can be used to fit complex functions or solve classification problems. The most typical structure of ANN consists of three layers—a labeled input layer, a hidden layer, and an output layer—as can be seen in Fig. 13, Structure of an ANN neural network.

In fact, ANNs benefit from a group of neurons and the relationships between them, which can differ from each other, by virtue of their assigned weights. ANN is considered as a feed-forward neural network, which means that there is one direction from input to output neurons. The information processing goes in this direction. Training algorithms, based on the backpropagation algorithm, perform learning and error-correction processes related to the input and output data layers. The ANN receives the input data to calculates the error value by assessing the target and output values. To minimize the error, ANN adjusts the weights of interconnections between neurons. The network keeps this process going, in order to obtain a logical minimum error. As our problem is a regression problem, the most commonly used loss function is the mean square error (MSE), which is the sum of the squares of the prediction data and the corresponding point error of the original data. The MSE can predefine the logical minimum error.

21 $$MSE\left( {y, y^{\prime } } \right) = \frac{{\mathop \sum \nolimits_{i = 1}^{n} \left( {y_{i} - y_{i}^{\prime } } \right)^{2} }}{n}$$

**Figure 14:**
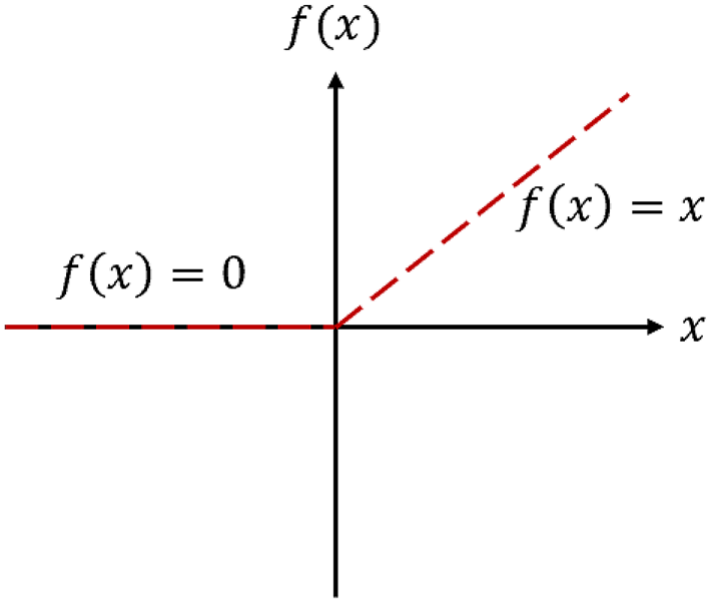
Activation function of neurons (*f*).

We used the rectified linear unit $$\left( {ReLU} \right)$$ function as the activation function of neurons ($$f)$$, as shown in Fig. 14.

22 $$ReLU\left( x \right) = \left\{ {\begin{array}{*{20}l} x \hfill & {if\;x > 0} \hfill \\ 0 \hfill & {if\;x \le 0} \hfill \\ \end{array} } \right.$$

The following mathematical equations describe a neuron $K$. In these equations, the output signal of the neuron is presented by $y_{k}$, activation function is represented by $f$, the linear output is $u_{k}$, the bias term is indicated by $b_{k}$, and input signals and interconnection weights are respectively denoted by $x_{i}$ and $w_{ki}$.

23 $$y_{k} = f\left( {u_{k} + b_{k} } \right),$$

24 $$u_{k} = \mathop \sum \limits_{i = 1}^{N} w_{ki} x_{i}$$

**Figure 15:**
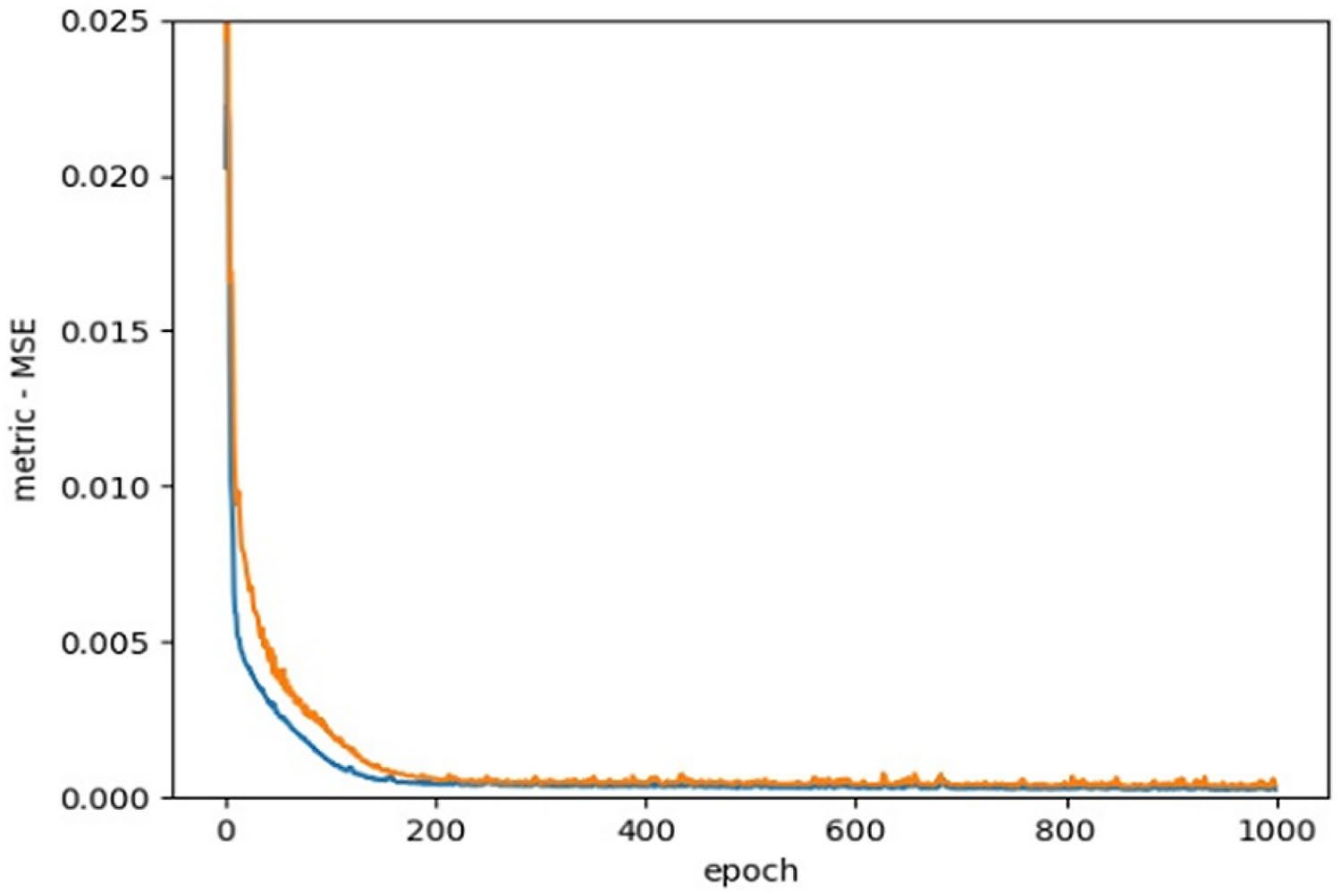
Loss function, MSE.

As Fig. 15, Loss function, presents—and based on previous studies— we use MSE as the loss function.

Adaptive Moment Estimation (ADAM) is an efficient method in the field of deep learning, which can be applied for first-order gradient-based optimization of stochastic objective functions by using momentum and adaptive learning rates to accelerate convergence. One of the most important principles in engineering research is the stochastic gradient-based optimization of parametric functions to maximize or minimize values with regards to function parameters. ADAM can work with sparse gradients and the significance of parameter updates is constant to gradient rescaling.

Empirical results have shown that the performance of ADAM is good in practice and better than other random optimization methods, such as AdaGrad, RMSProp, AdaDelta, SGDNesterov, and SFO (Sum-of-Functions Optimizer)^62–67^. In this research, we used the ADAM algorithm to optimize the model.

## Accuracy of LBS equation

### Methods of model evaluation

After obtaining the required multilinear regression equation, we needed to judge the goodness of fit of the regression equation, which was carried out through model evaluation.

For multi-linear regression, the most commonly used model evaluation indicator is likely the Mean Square Error (MSE):

25 $$MSE = \frac{1}{n}\mathop \sum \limits_{i = 1}^{n} \left( {y_{i} - \hat{f}\left( {x_{i} } \right)} \right)^{2}$$

This is the prediction at the *i*th observation point. If the response value of the predicted value is very close to the real response value, the MSE is very small; whereas, if there is a material difference between the predicted response value and the real response value, the MSE is very large. When the MSE is calculated from training data, it is called training MSE, but our general relationship is calculated for the test data (i.e., test error means square error). The appropriate model must be selected to minimize the test square error.

For multilinear regression, another commonly used model evaluation indicator is the multiple determination coefficient (Multiple Coefficient of Determination; R^2^). The multiple determination coefficient is a statistic that measures the fit of multiple regression equations and reflects the proportions explained by the estimated regression equations in the variance of the factor variable $y$, which is calculated as the proportion of the regression squares to the sum of total squares. The greater the goodness of fit, the higher the degree to which the argument interprets the cause variable, the higher the percentage of change caused by the argument to the total change, and the denser the observation points are near the regression line.

26 $$R2 - \frac{SSR}{{SST}} = 1 - \frac{SSE}{{SST}}$$

where $SST$ (total sum of squares) is the sum of squares, $SSR$ (regression sum of squares) is the sum of regression squares, and $SSE$ (error sum of squares) is the sum of residual squares.

27 $$SSR = \mathop \sum \limits_{i = 1}^{m} \left( {\hat{y}_{i} - \overline{y}} \right)^{2}$$

28 $$SSE = \mathop \sum \limits_{i = 1}^{m} \left( {y_{i} - \overline{y}} \right)^{2}$$

where $\hat{y}_{i}$ represents the model forecast value and $\overline{y}$ indicates the average of $y$.

### Comparison of LBS equations and ANN

**Figure 16:**
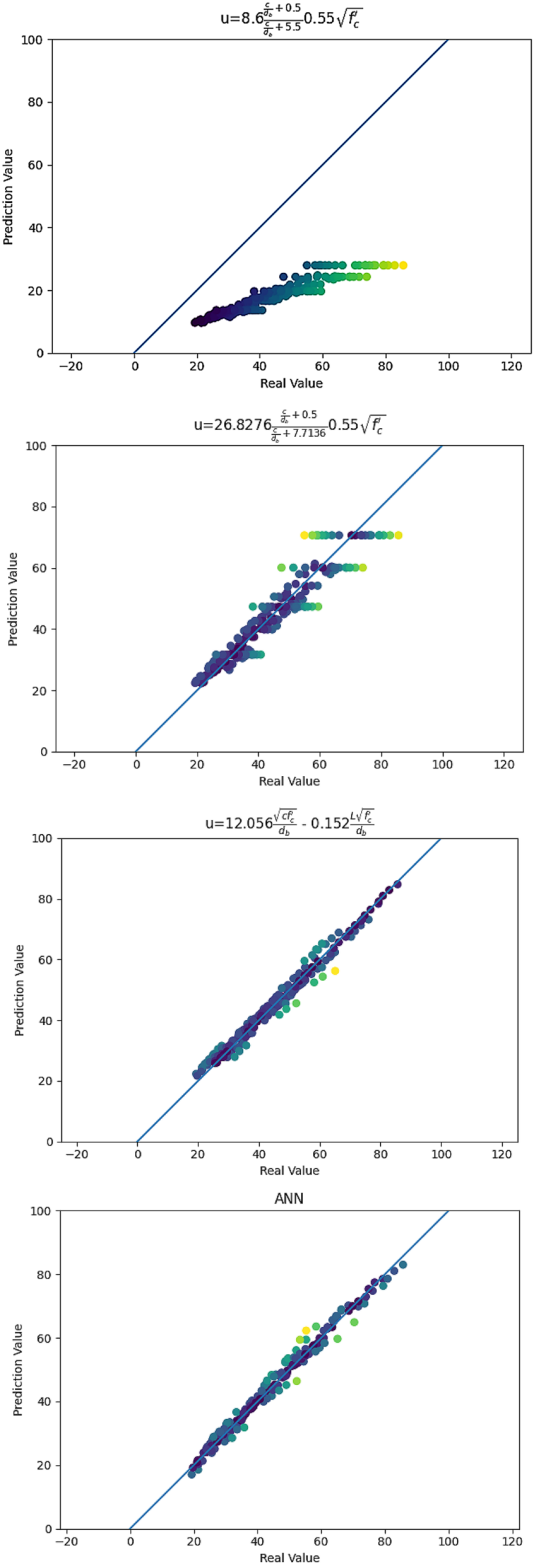
LBS equations and ANN accuracy.

Figure 16, LBS equations and ANN accuracy, represents a comparison between the accuracy of LBS Eqs. (1), (6), and (20) and the ANN. As can be seen, the predicted LBS values by Eq. (20) were closer to the middle line, thus justifying the precision of our proposed LBS Eq. (20). This accuracy may be related to the actual relationship between the effective parameters on local bond stress between UHPC and steel bars.

As Table 3 shows, from the MSE point of view, we can observe that Eq. (1) had an MSE of 803.784, which indicates a great deviation from reality. Equation (6) better fit the data but its MSE was 24.205, which was also large. Equation (20) and the ANN perfectly fit the data, with MSEs of 5.322 and 3.668, respectively.

**Table 3:** Evaluation of statistical parameters of ANN model.

Method	Evaluation Index					
	Training set		Test set		All set	
	*MSE*	*R*^2^	*MSE*	*R*^2^	*MSE*	*R*^2^
LBS Eq. (1)	781.956	− 2.557	1170.375	− 4.085	803.784	− 2.534
LBS Eq. (6)	22.747	0.897	44.129	0.808	24.205	0.894
LBS Eq. (20)	5.324	0.976	4.645	0.980	5.322	0.977
ANN	3.453	0.984	6.327	0.973	3.668	0.984

Considering the $R^{2}$ values, we can observe that the $R^{2}$ for Eq. (1) was − 2.534, with a negative value, indicating that the fitting result was unreliable and that the fitting function was seriously inconsistent with the data. The $R^{2}$ of Eq. (6) was 0.894 and, so, the fitting function was more in line with the data. Equation (20) and the ANN perfectly fit the data, with $R^{2}$ values of 0.977 and 0.984, respectively.

Table 4 provides a theoretical comparison between some of the LBS values obtained from our experimental pullout tests $\left( {Exp.} \right)$ and the numerical results $\left( {Num.} \right)$ in ABAQUS, as well as from Eqs. (1), (6), and (20). We chose some random specimens to investigate the precision of our proposed LBS equation. As can be seen from Table 4, there was good agreement between our test results and the LBS Eq. (20). In this table, $u_{test}$ represents the experimental and numerical pullout test results and $u_{5}$ represents that for the existing ACI equation, while $u_{10}$ and $u_{28}$ show the results from our LBS Eqs. (6) and (20).

**Table 4:** LBS results from Experimental tests, ABAQUS, and Eqs. (1), (6), and (20).

Specimen	$C$ $\left( {{\text{mm}}} \right)$	$d_{b}$ $\left( {{\text{mm}}} \right)$	$L\; \left( {{\text{mm}}} \right)$	$f_{c}^{\prime } \;\left( {{\text{MPa}}} \right)$	$u_{test} \;\left( {{\text{MPa}}} \right)$	$u_{1} \;\left( {{\text{MPa}}} \right)$	$u_{6} \;\left( {{\text{MPa}}} \right)$	$u_{20} \;\left( {{\text{MPa}}} \right)$	$\frac{{u_{1} }}{{u_{test} }}$	$\frac{{u_{6} }}{{u_{test} }}$	$\frac{{u_{20} }}{{u_{test} }}$
R12C2L2F155	24	12	24	155.1	57.54 _*Num*_	19.636	47.294	57.51	0.341	0.822	0.999
R12C4L2F155	48	12	24	155.1	82.89 _*Num*_	27.903	70.595	82.9	0.337	0.852	1
R12C4L3F155	48	12	36	155.1	80.81 _*Num*_	27.903	70.595	81.007	0.345	0.874	1.002
R14C1L1F155	14	14	14	155.1	37.74 _*Num*_	13.594	31.633	38.235	0.36	0.838	1.013
R14C3L1F155	42	14	14	155.1	68.52 _*Num*_	24.256	60.032	67.61	0.354	0.876	0.987
R14C1L2F155	14	14	28	155.1	36.52 _*Num*_	13.594	31.633	36.342	0.372	0.866	0.995
R16C2L1F155	32	16	16	155.1	52.83 _*Exp*_	19.636	47.294	51.191	0.372	0.895	0.969
R16C2L2F155	33	16	32	155.1	51.93 _*Exp*_	19.96	48.167	50.121	0.384	0.928	0.965
R16C4L1F155	64	16	16	155.1	76.11 _*Num*_	27.903	70.595	73.179	0.367	0.928	0.961
R18C3L2F155	54	18	36	155.1	55.262 _*Exp*_	24.256	60.032	57.51	0.439	1.086	1.041
R18C1L3F155	18	18	54	155.1	29.489 _*Exp*_	13.594	31.633	29.71	0.461	1.073	1.008
R18C4L3F155	72	18	54	155.1	61.97 _*Num*_	27.903	70.595	65.1	0.45	1.139	1.051
R20C4L3F155	80	20	60	155.1	57.56 _*Num*_	27.903	70.595	61.468	0.485	1.226	1.068
R20C2L4F155	40	20	80	155.1	38.18 _*Num*_	19.636	47.294	39.908	0.514	1.239	1.045
R20C4L2F155	80	20	40	155.1	58.84 _*Num*_	27.903	70.595	63.361	0.474	1.2	1.077

Based on the comparison provided in this table—and with regards to the last column— the accuracy and precision of our proposed LBS equation are evident. In this case, the LBS Eq. (20) can be reliably used to calculate the local bond stress between UHPC and steel bars.

## Conclusion

In this research, we studied the local bond stress between UHPC and steel bars. Nano-silica was used as an additive, in order to improve the compressive strength of the concrete. As the previous LBS equations were determined to not be precise enough to calculate the bond stress, we applied our test results—including pullout experiments and finite element software ABAQUS results—as input to an ANN. By passing mathematical calculations and comparing the data, we achieved a new method and proposed an accurate LBS equation.

1. According to our study outcomes, the ANN gave the most accurate results. Therefore, Eq. (20) was obtained and proposed to calculate the local bond stress between UHPC and steel bars.
2. The equations resulting from multiple linear regression could better express the effect of each variable on the LBS.
3. The proposed LBS equation outcomes were in good agreement with our pullout test and the ABAQUS results.
4. The multiple coefficient of determination (${R}^{2}$) of our proposed Eq. (20) was 0.977, while that for the ANN was 0.984. It can be seen that the proposed LBS Eq. (20) is precise enough for LBS calculation.

## References

[1] Jain AK, Mao J, Mohiuddin KM (1996). Artificial neural networks: A tutorial. Computer.

[2] Al-Alawi, S., Al-Badi, A., & Ellithy, K. An artificial neural network model for predicting gas pipeline induced voltage caused by power lines under fault conditions, COMPEL. *Int. J. Comput. Math. Electr. Electron. Eng*. **24**, 69–80 (2005).

[3] Oludolapo OA, Jimoh AA, Kholopane PA (2012). Comparing performance of MLP and RBF neural network models for predicting South Africa's energy consumption. J. Energy S. Afr..

[4] McCulloch WS, Pitts W (1943). A logical calculus of ideas immanent in nervous activity. Bull. Math. Biophys..

[5] Bala, R. & Kumar, D. Classification using ANN: A review. *Int. J. Comput. Intell. Res.* **13**(7), 1811–1820 (2017).

[6] Shang, C., Palmer, A., Sun, J., Chen, K.S., Lu, J. & Bi, J. “VIGAN: Missing view imputation with generative adversarial networks. In *2017 IEEE International Conference on Big Data (Big Data)* (pp. 766–775). IEEE. 10.1109/BigData.2017.8257992 PMC5813842 29457155

[7] Priyangga, H. Y. & Ruliandi, D. Application of pattern recognition and classification using artificial neural network in geothermal operation. In *Proceedings, 43rd Workshop on Geothermal Reservoir Engineering Stanford University, Stanford, California, February* 12–14, (2018).

[8] Ruiz-del-Solar, J., Loncomilla, P. & Soto, N. A survey on deep learning methods for robot vision 2018, arXiv preprint 1803.10862.

[9] He S, Lau RW, Liu W, Huang Z, Yang Q (2015). Super cnn: A super pixelwise convolutional neural network for salient object detection. Int. J. Comput. Vis..

[10] ThippeSwamy, R. D. K. Training feed forward neural network with back propogation algorithm. Int. J. Eng. Comput. Sci **6**(1) (2017).

[11] Li, S. & Deng, W. Deep facial expression recognition: A survey. arXiv preprint 1804.08348 (2018).

[12] Mahoor, B. H. M. Facial expression recognition using enhanced deep 3D convolutional neural networks. In *Proceedings of the IEEE Conference on Computer Vision and Pattern Recognition Workshops* (pp. 30–40), (2017).

[13] Seema, A. & Rajeshwar, D. Pattern recognition techniques: A review. *Int. J. Comput. Sci. Telecommun*. **3(**8) (2012).

[14] Schuurmans, D. & Zinkevich, M. Deep learning games. In *Advances in Neural Information Processing Systems (NIPS-16)* (2016).

[15] Hanchate, D. B., Nalawade, M., Pawar, M., Pophale, V. & Maurya, P. K. Vocal digit recognition using artificial neural network. In *2010 2nd International Conference on Computer Engineering and Technology (ICCET)* (Vol. 6, pp. V6–88). IEEE.

[16] Qazi, K. A., Nawaz, T., Mehmood, Z., Rashid, M. & Habib, H. A. A hybrid technique for speech segregation and classification using a sophisticated deep neural network. *PLoS ONE* **13**(3), e0194151 (2018). 10.1371/journal.pone.0194151 PMC5860734 29558485

[17] Mehta, R. & Arbel, T. RS-Net: Regression-segmentation 3D CNN for synthesis of full resolution missing brain MRI in the presence of tumours. 2018, arXiv preprint 1807.10972.

[18] El-Shahat, A. Advanced applications for artificial neural networks. BoD–Books on Demand (2018).

[19] Upadhyay, Y. Introduction to feedforward neural networks. *Towards Data Sci*. **7**. (2019).

[20] Svozil D, Kvasnicka V, Pospichal J (1997). Introduction to multi-layer feed-forward neural networks. Chemom. Intell. Lab. Syst..

[21] Peterson, R., Baldwin, A. & Schmidt, A. Comparative analysis of backpropagation algorithm against the ant colony optimization. *Int. J. Comput. Sci.* **1**(3) (2019).

[22] Huang, G. B., Zhu, Q. Y. & Siew, C. K. Extreme learning machine: A new learning scheme of feedforward neural networks. In *2004 IEEE International Joint Conference on Neural Networks Proceedings* (Vol. 2, pp. 985–990). IEEE (2004).

[23] Marović, I., Sušanj, I. & Ožanić, N. Development of ANN model for wind speed prediction as a support for early warning system. *Complexity* (2017).

[24] Sadowski L, Nikoo M (2014). Corrosion current density prediction in reinforced concrete by imperialist competitive algorithm. Neural Comput. Appl..

[25] Nikoo M, Zarfam P, Sayahpour H (2015). Determination of compressive strength of concrete using Self Organization Feature Map (SOFM). Eng. Comput..

[26] Yeh IC (1998). Modeling of strength of high performance concrete using artificial neural networks. Cem. Concr. Res..

[27] Nikoo, M., Torabian Moghadam, F. & Sadowski, A. Prediction of concrete compressive strength by evolutionary artificial neural networks. *Adv. Mater. Sci. Eng.* (2015). 10.1155/2015/849126.

[28] Gupta S (2013). Using artificial neural network to predict the compressive strength of concrete containing nano-silica. Civ. Eng. Architect..

[29] Suryadi, A., Triwulan, & Aji, P. Artificial neural network for evaluating the compressive strength of self compacting concrete*. J. Basic Appl. Sci. Res*. **1**(3), 236–241 (2011).

[30] Muthupriya P, Subramanian K, Vishnuram BG (2011). Prediction of compressive strength and durability of high performance concrete by artificial neural network. Int. J. Optim. Civ. Eng..

[31] Ahad Amini Pishro & Feng, X. Experimental and numerical study of nano-silica additions on the local bond of ultra-high performance concrete and steel reinforcing bar. *Civ. Eng. J*. 10.28991/cej-030962.

[32] Abellán J, Fernández J, Torres N, Núñez A (2020). Statistical optimization of ultra-high-performance glass concrete. ACI Mater. J..

[33] Abellán-García, J. Four-layer perceptron approach for strength prediction of UHPC. *Constr. Build. Mater*. (2020). 10.1016/j.conbuildmat.2020.119465.

[34] Abellán-García J, Fernández-Gómez J, Torres-Castellanos N (2020). Properties prediction of environmentally friendly ultra-high-performance concrete using artificial neural networks. Eur. J. Environ. Civ. Eng..

[35] Charhate, Sh., Subhedar, M. & Adsul, N. Journal of Soft Computing in Civil Engineering 2–3 (2018) 27–38. 10.22115/SCCE.2018.112140.1041.

[36] Khademi F, Behfarnia K (2016). Evaluation of concrete compressive strength using artificial neural network and multiple linear regression models. Int. J. Optim. Civ. Eng..

[37] Graybeal, B. & Tanesi, J. Durability of an ultrahigh-performance concrete. *J. Mater. Civil Eng. ASCE*, **19**(10), 848–854 (2007).

[38] Pyo, S., Alkaysi, M. & El-Tawil, S. Crack propagation speed in ultra high performance concrete (UHPC). *Constr. Build. Mater*. 109–118 (2016).

[39] Aitcin PC (2003). The durability characteristics of high performance concrete: a review. Cem. Concr. Compos..

[40] Yazici H (2007). The effect of curing conditions on compressive strength of ultra high strength concrete with high volume mineral admixtures. Build Environ..

[41] Abbas S, Nehdi ML, Saleem MA (2016). Ultra-high performance concrete: Mechanical performance, durability, sustainability and implementation challenges. Int. J. Concr. Struct. Mater..

[42] Shafiq N, Kumar R, Zahid M, Tufail RF (2019). Effects of modified metakaolin using nano-silica on the mechanical properties and durability of concrete. Materials.

[43] Zahid M, Shafiq N, Jalal A (2018). Investigating the effects of solarcure curing method on the compressive strength, microstructure, and polymeric reaction of fly ash based geopolymer. Constr. Build. Mater..

[44] Zahid M, Shafiq N, Nuruddin MF, Nikbakht E, Jalal A (2017). Effect of partial replacement of fly ash by metakaolin on strength development of fly ash based geopolymer mortar. Key Eng. Mater..

[45] Muhammad, Z., Nasir, S., Isa, M. H. & Gil, L. Statistical modeling and mix design optimization of fly ash based engineered geopolymer composite using response surface methodology. *Clean. Prod*. 194 (2018).

[46] Isfahani FT, Redaelli E, Lollini F, Li W, Bertolini L (2016). Effects of nano-silica on compressive strength and durability properties of concrete with different water to binder ratios. Adv. Mater. Sci. Eng..

[47] Ahad Amini Pishro & Feng, X. Experimental study on bond stress between ultrahigh performance concrete and steel reinforcement. *Civ. Eng. J.* **3**(12), 1235–1246 (2017).

[48] Feng X, Faisal R, Zahid M (2019). Experimental investigation and statistical modeling of frp confined ruc using response surface methodology. Civ. Eng. J..

[49] Bestgen JO, Cetin B, Tanyu BF (2016). Effects of extraction methods and factors on leaching of metals from recycled concrete aggregates. Environ. Sci. Pollut. Res..

[50] Rezaifar O, Hasanzadeh M, Gholhaki M (2016). Concrete made with hybrid blends of crumb rubber and metakaolin: Optimization using response surface method. Constr. Build. Mater..

[51] Ahad Amini Pishro, Feng, X., Ping, Y., Dengshi, H. & Shirazinejad, R. S. Comprehensive equation of local bond stress between UHPC and reinforcing steel bars. *Constr. Build. Mater*. **262**, 119942 (2020). 10.1016/j.conbuildmat.2020.119942.

[52] RILEM, Technical Recommendations for the Testing and Use of Construction Materials: RC6, Bond Test for Reinforcement Steed, 2. Pull-out Test, 1970.

[53] Jianxin, M. A. & Schneider, H. Properties of ultra-high-performance concrete. In: *LACER N 7, 6th International Symposium on Utilization of High Strength/High Performance Concrete, Germany* (2002).

[54] ASTM C234-91a, Standard Test Method for Comparing Concretes on the Basis of the Bond Developed with Reinforcing Steel, ASTM Annual Book of Standards, Section 4, Construction, Philadelphia.

[55] Foroughi-Asl A, Dilmaghani S, Famili H (2008). Bond strength of reinforcement steel in self-compacting concrete. Int. J. Civ. Eng..

[56] ABAQUS Analysis User’s Manual, Version 6.14-5 (2017).

[57] Al-Azzawi, A. A., Sultan, A. & Risan, A. K. H. Behavior of ultra-high performance concrete structures. *ARPN J. Eng. Appl. Sci.* (2011).

[58] ACI Committee 408, Bond and Development of Straight Reinforcing Bar in Tension (ACI 408R-03), American Concrete Institute, 2003.

[59] Darwin D, Zuo J, Tholen ML, Idun EK (1996). Development length criteria for conventional and high relative rib area reinforcing bars. ACI Struct. J..

[60] Zuo J, Darwin D (2000). Splice strength of conventional and high relative rib area bars in normal and high-strength concrete. ACI Struct. J..

[61] Esfahani MR, Rangan BV (1998). Local bond strength of reinforcing bars in normal strength and high-strength concrete (HSC). ACI Struct. J..

[62] Wang, S. & Manning, C. Fast dropout training. *Proceedings of the 30th International Conference on Machine Learning (ICML-13)*, pp. 118–126 (2013).

[63] Sutskever, I., Martens, J., Dahl, G. & Hinton, G. On the importance of initialization and momentum in deep learning. In *Proceedings of the 30th International Conference on Machine Learning (ICML-13)*, pp. 1139–1147 (2013).

[64] Sohl-Dickstein, J., Poole, B. & Ganguli, S. Fast large-scale optimization by unifying stochastic gradient and quasi-newton methods. In *Proceedings of the 31st International Conference on Machine Learning (ICML-14)*, pp. 604–612 (2014).

[65] Moulines, E. & Bach, F. R. Non-asymptotic analysis of stochastic approximation algorithms for machine learning. *Advances in Neural Information Processing Systems*, pp. 451–459 (2011).

[66] Graves, A., Mohamed, A. & Hinton, G. Speech recognition with deep recurrent neural networks. In *2013 IEEE International Conference on Acoustics, Speech and Signal Processing (ICASSP)* pp. 6645–6649. IEEE (2013).

[67] Deng, L., Li, J., Huang, J.-T., Yao, K., Yu, D., Seide, F., Seltzer, M., Zweig, G., He, X.,& Williams, J. *et al.* Recent advances in deep learning for speech research at microsoft. *ICASSP* 2013 (2013).

